# The contribution of smoking-attributable mortality to differences in mortality and life expectancy among US African-American and white adults, 2000–2019^1^

**DOI:** 10.4054/demres.2022.46.31

**Published:** 2022-05-12

**Authors:** Brian L. Rostron, Brittny C. Davis Lynn, Cindy M. Chang, Chunfeng Ren, Esther Salazar, Bridget K. Ambrose

**Affiliations:** 2 US Food and Drug Administration, United States.; 3 US Food and Drug Administration, United States.

## Abstract

**BACKGROUND:**

The role of smoking in racial disparities in mortality and life expectancy in the United States has been examined previously, but up-to-date estimates are generally unavailable, even though smoking prevalence has declined in recent decades.

**OBJECTIVE:**

We estimate the contribution of smoking-attributable mortality to observed differences in mortality and life expectancy for US African-American and white adults from 2000–2019.

**METHODS:**

The indirect Preston–Glei–Wilmoth method was used with national vital statistics and population data and nationally representative never-smoker lung cancer death rates to estimate the smoking-attributable fraction (SAF) of deaths in the United States by sex-race group from 2000–2019. Mortality rates without smoking-attributable mortality were used to estimate life expectancy at age 50 (*e*_50_) by group during the period.

**RESULTS:**

African-American men had the highest estimated SAF during the period, beginning at 26.4% (95% CI:25.0%–27.8%) in 2000 and ending at 12.1% (95% CI:11.4%–12.8%) in 2019. The proportion of the difference in *e*_50_ for white and African-American men that was due to smoking decreased from 27.7% to 14.8%. For African-American and white women, the estimated differences in *e*_50_ without smoking-attributable mortality were similar to observed differences.

**CONCLUSIONS:**

Smoking continues to contribute to racial disparities in mortality and life expectancy among men in the United States.

**CONTRIBUTION:**

We present updated estimates of the contribution of smoking to mortality differences in the United States using nationally representative data sources.

## 1. Introduction

Cigarette smoking remains the leading preventable cause of disease and death in the United States (Danaei et al. 2009), despite declines in prevalence in recent decades. The Centers for Disease Control and Prevention (CDC) estimates that smoking is responsible for about 480,000 premature deaths in the United States each year (National Center for Chronic Disease Prevention and Health Promotion Office 2014).

Despite having had a smoking prevalence similar to that of their white counterparts in recent years (Garrett et al. 2011) and fewer reported cigarettes smoked per day (Muscat, Richie, and Stellman 2002), African-Americans have experienced a higher burden of smoking-related disease and death than white Americans (Ho and Elo 2013). This disparity results in part from a higher smoker prevalence among African-Americans in previous decades, especially for men (Garrett et al. 2011). African-Americans also continue to experience overall disadvantages in mortality and life expectancy. In 2018, at age 50 non-Hispanic Black men could expect to live 3.1 years less than white men, and among women the difference was 1.9 years (Arias and Xu 2020). In 1999–2001 the difference was 4.0 years for men and 3.0 years for women (Arias, Rostron, and Tejada-Vera 2010).

Because of these racial disparities in smoking-related and overall mortality, it is informative to have contemporary estimates of the effect of smoking on adult mortality and life expectancy differentials for US African-Americans and whites. Ho and Elo (2013) previously used an indirect method to estimate the contribution of smoking to differences in mortality and life expectancy for these groups during 1980–2005. This method provides reliable estimates of smoking-attributable mortality over time with a consistent methodology using readily available population and vital statistics data and captures smoking-attributable mortality from lung cancer and other causes. This analysis updates and extends such estimates to the period 2000–2019 using nationally representative never-smoker lung cancer death rates. Ultimately, this provides timely estimates of the mortality effects of smoking by sex and race in the United States and shows differences for these groups.

## 2. Methods

Smoking-attributable mortality by sex and race group for those 50 years and older in the United States was estimated using the indirect PGW method presented by Preston, Glei, and Wilmoth (2010) and further developed by Fenelon and Preston (2012). This method assumes that excess lung cancer mortality observed in a population is attributable to smoking. It also uses lung cancer mortality to estimate smoking-attributable mortality from other causes. Specifically, the PGW method calculates the smoking-attributable fraction (SAF) of deaths from lung cancer, *A*_*L*_, by sex and age group as:

(1) $$A_{L}=\frac{M_{L}-M_{L}^{*}}{M_{L}}$$

where *M*_*L*_ is the observed lung cancer death rate in a population and $M_{L}^{*}$ is the death rate among lifetime never-smokers in a study population. Preston, Glei, and Wilmoth (2010) and Fenelon and Preston (2012) used never-smoker death rates from the American Cancer Society’s Cancer Prevention Study II (CPS-II), a large non-representative cohort study. Lariscy, Hummer, and Rogers (2020) subsequently estimated lung cancer death rates for lifetime never-smokers using nationally representative NHIS data from 1985 to 2014 with mortality follow-up through 2015 (NHIS-LMF), which were used here.

The PGW method then uses the estimated association between lung cancer death rates and death rates from all other causes to estimate smoking-attributable mortality from these other causes. Preston, Glei, and Wilmoth (2010) conducted a negative binomial regression analysis of the relationship between mortality from lung cancer and other causes in 20 high-income countries from 1950 to 2006 to obtain coefficients expressing this relationship. Fenelon and Preston (2012) conducted a similar analysis using US data from all 50 states from 1996 to 2004 to obtain coefficients for ages 50–84 years, which were used here. The SAF for causes other than lung cancer, *A*_*o*_, is calculated as:

(2) $$A_{O}=\frac{e^{B_{L}^{\prime }\left(M_{L}\right)}-e^{B_{L}^{\prime }\left(M_{L}^{*}\right)}}{e^{B_{L}^{\prime }\left(M_{L}\right)}}$$

where $B_{L}^{\prime }$ are the sex- and age-specific coefficients expressing the relationship between lung cancer mortality and mortality from other causes. The SAF for all deaths is then calculated as the proportion of deaths that are attributable to smoking across age groups:

(3) $$\text{SAF}=\frac{\sum_{\text{age}}\left(A_{L}D_{L}+A_{O}D_{O}\right)}{\sum_{\text{age}}D}$$

where *D*_*L*_, *D*_*o*_, and *D* are the numbers of deaths from lung cancer, other causes, and all causes, respectively. All values for SAFs are presented as percentages in this study.

This analysis estimated SAFs by sex (male and female) and race (Black or African-American and White) group in the United States from 2000 to 2019 using these methods. National bridged-race mortality and population data were obtained by five-year age groups for ages 50 to 84 and for ages 85 and older from the CDC’s WONDER website (2021). Deaths with lung cancer as the underlying cause were identified using the International Classification of Diseases, Revision 10 codes C33–C34, consistent with the data sources used with the PGW method (Lariscy, Hummer, and Rogers 2020). SAFs are presented for ages 50 to 84 using Fenelon and Preston’s coefficients, consistent with the analysis by Lariscy, Hummer, and Rogers (2020). Confidence intervals (CIs) were estimated with 10,000 simulations conducted using a method presented by Preston, Glei, and Wilmoth (2010) with a Poisson distribution for lung cancer and all-cause death rates and a multivariate normal distribution for the beta coefficients. For purposes of comparison, SAFs calculated using CPS–II never-smoker lung cancer death rates (Thun et al. 1997) are presented for 2019. Similarly, SAFs for ages 50 and older were calculated for this year using the Fenelon and Preston coefficient for ages 80–84 for 85 and older, consistent with the presentation by Ho and Elo (2013).

Observed all-cause death rates and rates removing estimated smoking-attributable mortality were then used to construct life tables beginning at age 50 using standard techniques (Arias 2012). These life tables were used to compare differences in life expectancy at age 50, *e*_50_, between African-Americans and whites by sex, with and without smoking-attributable mortality. Again, the coefficient from Fenelon and Preston for ages 80–84 was used for 85 and older to calculate *e*_50_, similar to the approach used by Ho and Elo (2013). Detailed calculations and results are presented as supplementary material.

## 3. Results

**Figure 1:**
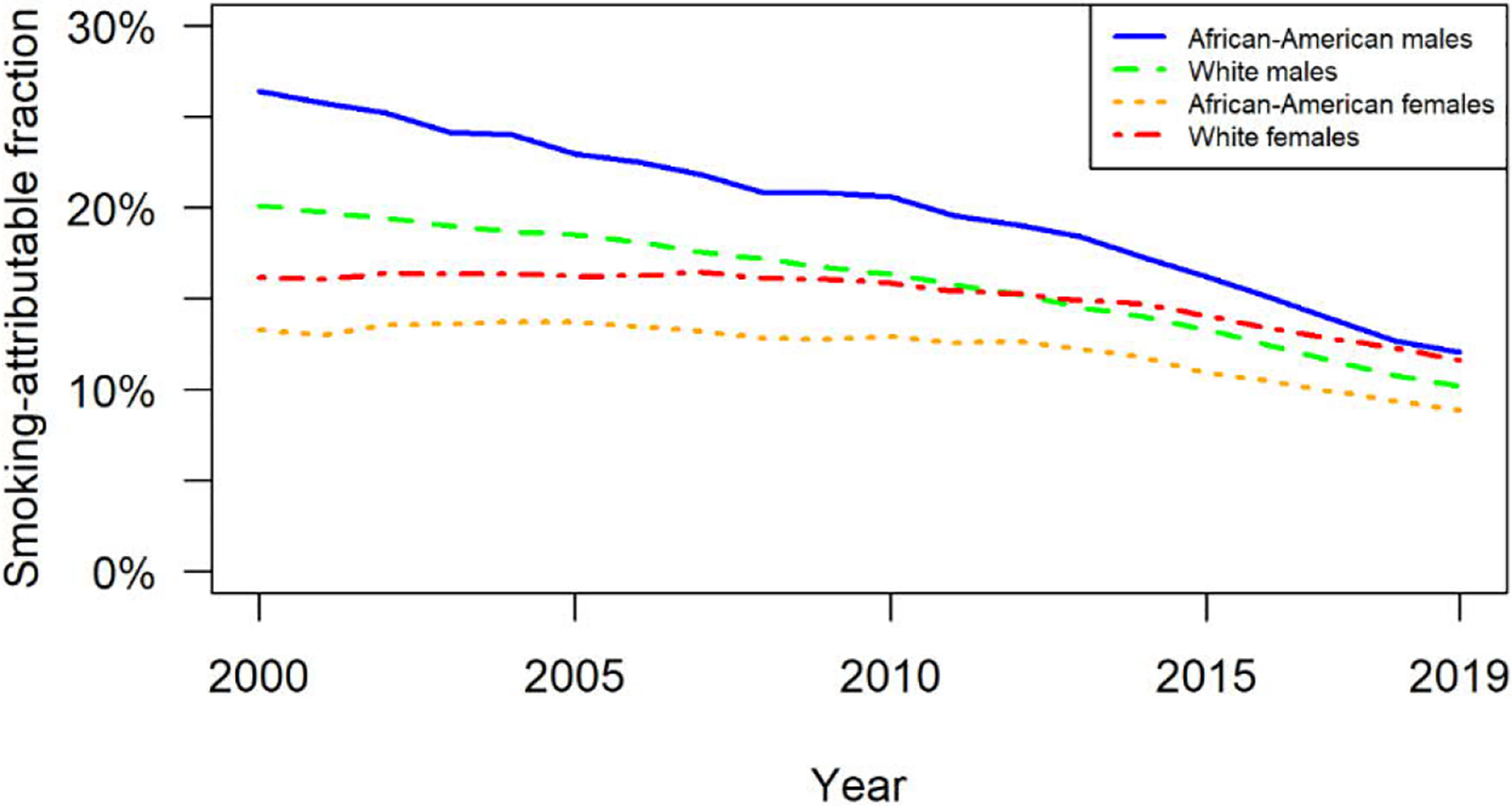
Smoking-attributable fractions of deaths in the United States over time, ages 50–84, by sex and race group

Figure 1 presents estimates of SAFs by sex and race group for the United States from 2000 to 2019. African-American men consistently had the highest SAF of the four groups, beginning the period at 26.4% (95% CI:25.0%–27.8%) compared to 20.1% (95% CI:18.9%–21.4%) for white men. By 2019 the SAFs were 12.1% (95% CI:11.4%–12.8%) and 10.2% (95% CI:9.6%=10.8%), respectively. African-American women generally had the lowest SAF of the groups, starting at 13.3% (95% CI:11.8%–14.8%) in 2000 compared to 16.2% (95% CI:–14.3%–17.9%) for white women. In 2019 the SAFs were 8.9% (95% CI:7.9%–9.9%) and 11.6% (95% CI:10.4%–12.9%) for these groups. In general, the SAFs for the two female groups were relatively constant during the first ten years of the period before decreasing more substantially in later years. The SAFs for the male groups consistently declined during the period.

Table 1 includes estimates for 2019 calculated using alternative specifications. Removing Hispanics slightly increased SAFs, particularly for whites. SAFs calculated using CPS-II never-smoker death rates were somewhat higher than those calculated using more recent NHIS-LMF rates. SAFs calculated for ages 50 and older were somewhat lower for every group.

Figure 1 and Table 2 show the estimated difference in *e*_50_ between African-Americans and whites in the United States from 2000 to 2019 with and without smoking-attributable mortality. Among men, observed *e*_50_ was 28.25 years among whites and 24.32 years for African-Americans in 2000, for a deficit in life expectancy of 3.93 years. In the absence of smoking-attributable mortality the difference in *e*_50_ would have been 2.84 years, for a reduction of 27.7% in the life expectancy deficit between white and African-American men. The contribution of smoking-attributable mortality to the difference in *e*_50_ by race slowly decreased among men during the period. By 2019 the observed difference was 2.52 years and the adjusted difference without smoking-attributable mortality was 2.15, for a decrease in the life expectancy deficit of 14.8%.

Among women, observed *e*_50_ was 29.25 among African-Americans and 32.12 among whites in 2000, for a life expectancy deficit of 2.87 years. Without smoking-attributable mortality the difference in *e*_50_ would have been 2.96 years, for an increase in the life expectancy deficit of 2.9%. The estimated effect of smoking-attributable mortality on the difference in *e*_50_ by race remained generally similar among women during the period. In 2019 the observed difference in *e*_50_ was 1.28 years and the adjusted difference without smoking-attributable mortality was 1.39, for an increase of 8.9% in the life expectancy deficit.

## 4. Discussion

This study has found that cigarette smoking has consistently contributed to differences in mortality and life expectancy by race in the United States over the last two decades. Use of an established indirect method has shown that smoking has been responsible for a substantial portion of deaths across sex and race groups, with African-American men consistently having the highest proportion of deaths due to smoking. It is estimated that among men, smoking-attributable mortality caused almost 28% of the reduction in the difference in life expectancy at age 50 for African-Americans compared to whites in 2000 and approximately 15% of the reduction in 2019. Among women the contribution of smoking to differences in mortality and life expectancy by race was somewhat smaller during the period. The estimated difference in *e*_50_ for white and African-American women without smoking-attributable mortality would have been about 3% greater in 2000 and 9% greater in 2019.

The results in this study are generally similar to comparable estimates from an earlier study. Ho and Elo (2013) examined the role of smoking in Black-white mortality differences in the United States from 1980 to 2005 using the PGW method. Among men, they found that the contribution of smoking to racial differences in life expectancy peaked around 1984, representing almost 50% of the difference in *e*_50_ between Blacks and whites, and then generally declined. They estimated that smoking accounted for 22.7% of the racial difference in *e*_50_ in 2005 using CPS-II never-smoker lung cancer death rates, compared to an estimate of 20.8% in this study. The comparability of estimates for females is even greater. Ho and Elo (2013) estimated that the adjusted difference in *e*_50_ for white and Black women in 2005 was 0.06 years, compared to a difference of 0.07 here.

These results are also consistent with previously observed trends in smoking prevalence and smoking-attributable mortality. As noted, smoking prevalence has generally been decreasing in the United States for several decades. For example, US adult smoking prevalence in 1965 was about 42% (Wang et al. 2018), but in 2000 it was approximately 23% (Centers for Disease Control and Prevention 2002) and by 2019 it was 14% (Cornelius et al. 2020). Moreover, in a report on US adult smoking prevalence by sex and race/ethnicity group from 1965 to 2008, Garrett et al. (2011) showed that smoking prevalence among men was about 10 percentage points higher for non-Hispanic Blacks than whites at the beginning of the period but had declined to similar levels by its end, consistent with the subsequent decreases in the contribution of smoking to differences in life expectancy in this study. Other factors contributing to differences in smoking-attributable mortality by race among men could include differences in smoking duration, nicotine metabolism, and toxicant exposure (Ho and Elo 2013). Among women, non-Hispanic Blacks had a smoking prevalence that was generally at or slightly below the level for whites during much of this time, again consistent with the trends outlined here.

Many factors other than smoking could account for the remaining mortality differences by race, including access to health care, differences in treatment, other health behaviors, environmental exposures, and the effects of economic and social inequality (Orsi, Margellos-Anast, and Whitman 2010). The racial difference in estimated life expectancy without smoking is now greater among men than women. African-American men have been observed to have particularly high death rates from certain causes, including homicide, AIDS, and diabetes (Pathak 2018).

This study is subject to some limitations. The indirect PGW method has been well-validated (Fenelon and Preston 2012) but does include various assumptions. For example, lung cancer mortality is assumed to have an established relationship with smoking-attributable mortality from other causes, based on a regression analysis. This study used coefficients from an analysis of mortality in the United States from 1996 to 2004, but mortality patterns for lung cancer and other causes could change over time or vary by race group. Some research has found similar lung cancer risks for African-Americans and whites with similar smoking histories (Stellman et al. 2003). The method also calculated lung cancer mortality attributable to smoking with reference to never-smoker death rates. Again, we used nationally representative US rates from a recent period, but they were not calculated for specific races. Smoking-attributable mortality before age 50 was not considered here, although the effect on differences in mortality and life expectancy should be limited. A previous study of Finnish data extended the PGW method to age 30 but found that different specifications had little effect on life expectancy estimates because smoking-attributable mortality at younger ages is very low (Martikainen et al. 2014). We estimated racial mortality differences for African-American and white adults overall, consistent with previous research on this topic (Ho and Elo 2013), for purposes of comparability and because of some issues with Hispanic ethnicity reporting on death certificates, although their effect on overall mortality estimates may be limited (Arias et al. 2008). As noted, the restriction to non-Hispanics made a relatively small difference in estimates of smoking-attributable mortality by race. Hispanics tend to have relatively low smoking prevalence in the United States (Cornelius et al. 2020) and most report ‘white’ or ‘some other race’ as their race category (Liebler et al. 2017), so their exclusion would tend to increase smoking-attributable mortality among white adults somewhat. Additional analyses could consider the effect of smoking-attributable mortality on mortality and life expectancy differentials for other races in the United States, such as Asian-Americans. Finally, life tables were constructed with standard methods consistent with those used to construct the US life tables, but we did not implement the entire detailed methodology used to construct those tables, including adjustment for race and Hispanic origin misreporting and use of Medicare data at older ages (Arias 2012).

Results in this study confirm continuing differentials in mortality outcomes in the United States due to previous smoking exposure, especially among African-American men. Differences in life expectancy narrowed somewhat between 2000 and 2019, but in 2019 the life expectancy of African-American men at age 50 was still estimated to be 2.52 years fewer than that of whites, of which 0.37 years were due to smoking. The US Food and Drug Administration has announced plans to ban characterizing flavors in menthol cigarettes (Knopf 2021), which are disproportionately used by African-Americans and other vulnerable populations (Lawrence et al. 2010). For example, 29% of non-Hispanic white smokers aged 12 years and older reported using menthol cigarettes in the 2012–2014 National Survey on Drug Use and Health, compared to 85% of non-Hispanic Black smokers (Villanti et al. 2016). This measure should further reduce racial disparities in mortality and life expectancy by reducing smoking initiation and increasing cessation, particularly among African-Americans (Levy et al. 2021).

## Supplementary Material

Data and Calculations

Life Tables

## Figures and Tables

**Figure 2:**
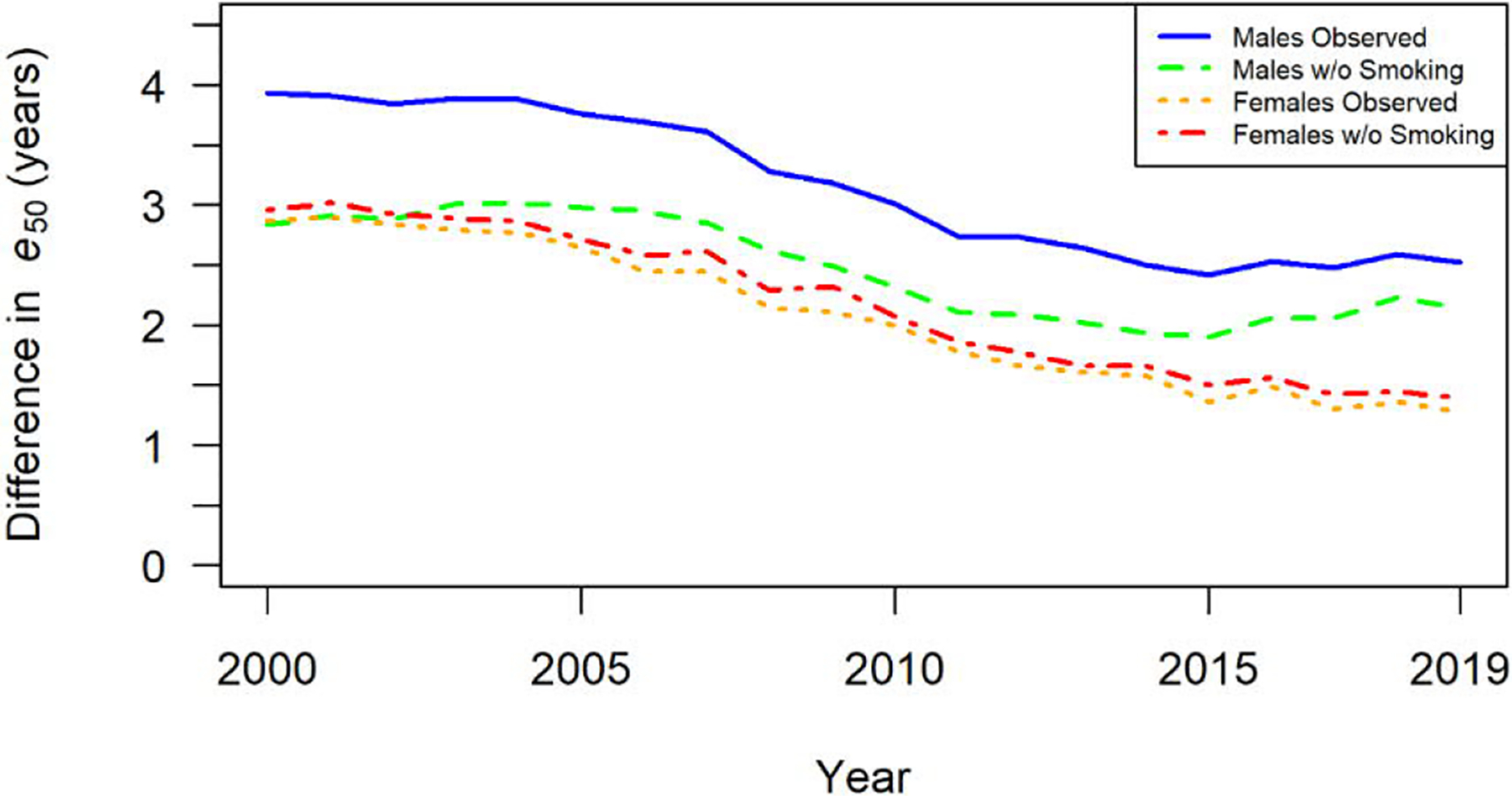
Differences in life expectancy at age 50, *e*_50_, for US African-Americans and Whites with (observed) and without smoking-attributable mortality, by sex and year

**Table 1:** Smoking-attributable fractions of deaths (95% CIs in parentheses) in the United States in 2019 with alternative specifications, by sex and race group

	Restricted to non-Hispanics (ages 50–84)	Using CPS-II never-smoker death rates (ages 50–84)	For ages 50+
African-American males	12.5% (11.8%–13.3%)	14.0% (13.3%–14.8%)	11.2% (10.4%–11.9%)
White males	10.9% (10.3%–11.6%)	12.1% (11.5%–12.8%)	8.9% (8.1%–9.6%)
African-American females	9.2% (8.1%–10.2%)	10.0% (9.0%–11.1%)	7.8% (6.7%–8.9%))
White females	12.6% (11.3%–13.9%)	12.9% (11.6%–14.2%)	9.5% (8.0%–10.9%)

**Table 2:** Life expectancy at age, *e*_50_, for US African-Americans and whites with (observed) and without (adjusted) smoking-attributable mortality (SAM), by sex and year

	2000		2005		2010		2015		2019	
	Female	Male	Female	Male	Female	Male	Female	Male	Female	Male
Observed *e*_50_, African-Americans	29.25	24.32	30.13	25.42	31.62	26.99	32.30	27.75	32.76	27.95
Observed *e*_50_, Whites	32.12	28.25	32.78	29.18	33.62	30.00	33.67	30.18	34.04	30.47
Adjusted *e*_50_,^1^ African-Americans	30.67	27.52	31.60	28.13	33.07	29.37	33.53	29.64	33.77	29.35
Adjusted *e*_50_,^1^ Whites	33.63	30.37	34.32	31.11	35.14	31.69	35.03	31.54	35.16	31.50
Observed Difference in *e*_50_	2.87	3.93	2.65	3.76	2.00	3.01	1.36	2.42	1.28	2.52
Adjusted Difference in *e*_50_	2.96	2.84	2.72	2.98	2.07	2.32	1.50	1.90	1.39	2.15
% Change in Difference in *e*_50_ without SAM	2.9%	−27.7%	2.6%	−20.8%	3.5%	−22.8%	9.7%	−21.5%	8.9%	−14.8%

*Note*: Adjusted e50 represents life expectancy at age 50 calculated without smoking-attributable mortality estimated using the PGW indirect method as described in the text.
